# Reserves Protect against Deforestation Fires in the Amazon

**DOI:** 10.1371/journal.pone.0005014

**Published:** 2009-04-08

**Authors:** J. Marion Adeney, Norman L. Christensen, Stuart L. Pimm

**Affiliations:** Nicholas School of the Environment, Duke University, Durham, North Carolina, United States of America; University of Sheffield, United Kingdom

## Abstract

**Background:**

Reserves are the principal means to conserve forests and biodiversity, but the question of whether reserves work is still debated. In the Amazon, fires are closely linked to deforestation, and thus can be used as a proxy for reserve effectiveness in protecting forest cover. We ask whether reserves in the Brazilian Amazon provide effective protection against deforestation and consequently fires, whether that protection is because of their location or their legal status, and whether some reserve types are more effective than others.

**Methodology/Principal Findings:**

Previous work has shown that most Amazonian fires occur close to roads and are more frequent in El Niño years. We quantified these relationships for reserves and unprotected areas by examining satellite-detected hot pixels regressed against road distance across the entire Brazilian Amazon and for a decade with 2 El Niño-related droughts. Deforestation fires, as measured by hot pixels, declined exponentially with increasing distance from roads in all areas. Fewer deforestation fires occurred within protected areas than outside and the difference between protected and unprotected areas was greatest near roads. Thus, reserves were especially effective at preventing these fires where they are known to be most likely to burn; but they did not provide absolute protection. Even within reserves, at a given distance from roads, there were more deforestation fires in regions with high human impact than in those with low impact. The effect of El Niño on deforestation fires was greatest outside of reserves and near roads. Indigenous reserves, limited-use reserves, and fully protected reserves all had fewer fires than outside areas and did not appear to differ in their effectiveness.

**Conclusions/Significance:**

Taking time, regional factors, and climate into account, our results show that reserves are an effective tool for curbing destructive burning in the Amazon.

## Introduction

Tropical moist forests hold the majority of species and are shrinking by more than 1 million km^2^/decade [1]. Reserves —broadly defined — are the principal means to conserve these forests and the biodiversity within them [2]. Do reserves actually protect natural ecosystems and their biodiversity? This may not have a simple answer, for there are many confounding factors. Even if reserves do work, are they simply protected *de facto* by their isolation or terrain, or *de jure*, because protected status does indeed provide added benefit?

We accept that reserves may be in suboptimal places to protect biodiversity [3], may not prevent hunting [4], and may be too small to maintain viable populations of many species [1]. That said, reserves that protect forest cover are a necessary, if not sufficient, criterion for protecting biodiversity. Credible, global assessments of reserve effectiveness are few [5]. Recently, we showed that for the Amazon and Congo basins, (which retain large areas of forests) and West Africa and the coastal forests of Brazil (which do not), reserves retain substantial natural forest cover [6]. For the Amazon and the Congo, so do most areas outside of the reserves.

More detailed regional studies suggest that reserves range in effectiveness — from those that do not work at all [7], [8] to those that work well [9]. In the Brazilian Amazon (a legally defined area), reserves have less deforestation [10] and fire [11] than do unprotected areas. For this region, the answer, then, is apparently that reserves do work. We will argue that these questions need a more detailed analysis than is presently available.

Whether reserves work is a question of considerable importance, regionally and internationally. A history of massive deforestation linked to large-scale infrastructure projects (notably roads) in the Brazilian Amazon, and government plans for more such projects, has spawned debate about ways to avoid repeating past trends [12]–[14]. Global concern about climate change and substantial carbon released from forest cutting and burning has added international impetus, including new funding mechanisms. Recently, Brazilian president Luiz Inácio Lula da Silva, created an international Amazon fund, which is hoped will raise up to 21 billion dollars, to allow countries, companies and non-governmental organizations to help pay for conservation, sustainable development, and scientific research in the Amazon [15]. The plan to repave Brazil's highway BR-319 from Manaus to Porto Velho, which would link the relatively intact central Amazon with centers of deforestation in the south, is another pressing issue with important implications for forest preservation. Reserve creation around BR-319 is part of the ongoing discussion about how to avoid massive land grabbing and deforestation that has accompanied other roads [16], [17].

### The Amazon, deforestation, and fires

In the Amazon, deforestation and fire are inextricably linked. Satellite-detected “hot pixels” are a good proxy for deforestation fires, and can thus effectively tell us whether reserves are protecting forest cover. In addition to biodiversity concerns, deforestation fires indicate that biomass has rapidly been released as carbon to the atmosphere, another important measure of reserve effectiveness. Our analyses concentrate on fires as a measure of human impact and on the ability of reserves to mitigate it.

Studies that have looked at fire in the Amazon [11], [18]–[21] are an important start. Nepstad et al. [21] did assert that reserves inhibit deforestation and fires at their borders. However, they did not examine the number or spatial arrangement of fires throughout reserves. They used the ratio of fire density in 20 km wide buffers inside and outside reserve borders to gauge protection inside a reserve against disturbance just outside. Although these methods indicate that fire stops at reserve borders, they tell us nothing about what is going on inside of a reserve (if, for example, a reserve has a road through it). They used a limited sample of reserves, as they did not include state protected areas, and they examined 4 km^2^ resolution GOES (Geostationary Operational Environmental Satellites) hot pixels for 1998 only. Finally, because Nepstad et al. [21] used only hot pixels detected at mid-day, they included pasture/agricultural fires that take place on deforested land. Nepstad et al. also did not explicitly consider distance from road, region, any climatic factors, or year to year variation. This and other studies (mentioned above) are generally geographically or temporally restricted and none has explicitly looked at fire patterns inside reserves.

We start by considering the factors that affect fire incidence. Because there is large year-to-year variation in climate and in fires [18], we examined fires over a decade, using remote-sensing products that mainly register deforestation fires. To account for the substantial gradient of rainfall and human impact [22] that influences pressures on reserves across the Amazon, we designated two distinct regions, which we examined separately. Because roads are so important [11], [20], [23]–[25], we modeled deforestation fires with increasing distance to roads across the entire area of reserves, not just at their borders.

In short, we ask three key questions: (1) Do reserves actually protect Amazonian forests from deforestation and consequently fires? (2) Is protection *de facto*, a consequence of reserve location (in remote places, for example), or *de jure*, because legal protections are respected? (3) Are some reserve types more effective than others in preventing deforestation fires? We recognize that there will be confounding factors: (a) Given that severe droughts remove moisture limitations and thus promote the spread of fires, do different kinds of reserves offer varying levels of protection in El Niño Southern Oscillation (ENSO) years? (b) Do reserves prevent deforestation fires even when human access is possible through road networks? (c) Finally, given these other factors, do the answers vary from place to place across the Amazon?

### The effects of roads and reserves

In the Amazon, roads are the major conduits for deforestation and accompanying fires [12], [23], [25], [26]. Because of the access they provide, roads may cause deforestation to increase even in neighboring roadless areas [12], [24]. Disturbed or fragmented forests near roads are vulnerable to both “leaked fires” used in land management and to accidental fires resulting from increased ignition sources [18]. Whatever the cause, the higher fuel loads and an open canopy in a forest already subject to understory fire greatly increase the chances for a hot deforestation fire [27], such as those visible in satellite imagery.

Theoretically, reserves may halt fire because of restrictions on land use (forests are less disturbed and fire is not used for management) or because of restricted access (fewer roads and fewer ignition sources). Different types of reserves in Brazil allow different land uses [28]. Strictly protected parks allow no habitation or clearing. Limited-use areas may allow selective logging, extraction of forest products, agriculture, and even private property within reserve boundaries. Indigenous people of many languages, cultures, and values control indigenous lands and sometimes protect them from logging, mining and illegal hunting [29].

Whatever the mechanism, reserves clearly limit road building, deforestation and fire in many highly affected areas [21], [30]. However, reserves may have fewer deforestation fires because they have fewer roads bisecting them then do adjacent unprotected areas. Whether reserves that do have roads also prevent deforestation and fire along those roads, and whether some reserve types do this better than others, have not been examined. In addition to reserve type, political and economic factors, including road paving, infrastructure projects, and beef and soy prices influence the likelihood of deforestation fires differently in different regions [11], [13]. Finally, drought may drive fire patterns [31] regardless of a reserve's status.

### Regional and year-to-year differences in climate

Climate patterns produce different spatial and temporal patterns of drought in different regions of the Amazon [19], [22]. ENSO commonly causes drought in the tropics [32]. ENSO-related droughts [22] and temperature changes [33] are strongest in the northern Amazon; however, these areas are also protected from fire by high background rainfall (up to 4000 mm annually) [34] and remoteness (fewer roads and people result in fewer ignitions) [19].

The leading edge of development in the Brazilian Amazon forms an arc from the southwestern to the southeastern Amazon. Here, seasonally dry forests (1500–2000 mm of rain annually) [34] become vulnerable to fires when drought further lengthens the dry season [22]. Both ENSO and the Atlantic Multidecadal Oscillation [35] can increase dry-season length in the southwest Amazon, as occurred in both the 1997–1998 ENSO-related drought [36] and the 2005 Amazon drought (which resulted in many fires) [37]. This area is more accessible from the populated south and is conducive to farming and cattle ranching, increasing incentives to clear land [22], [38]. Fire used in agriculture results in more potential ignitions [39]. Dry-season severity also increases fire frequency [31], as “leaked” understory fires escape into drought-stressed forests with higher fuel loads (from disturbed canopies or dead organic matter) [19]. Deforestation fires, while probably exacerbated by drought, are driven by policy and economic factors [40]. Finally, drought exacerbates positive feedbacks in which fires reduce rainfall and increase the chance of future fires [31]. Some climate models predict increased warming and decreasing soil water in the eastern Amazon over the next century [41]. Such changes could greatly increase fire risk across huge areas of the Amazon [19], [31].

Because processes affecting fire differ between these regions, we divided the Amazon using relevant political and geographical boundaries and analyzed forest areas in the two regions separately.

## Materials and Methods

### Data sources

We used 3 remotely-sensed data sources to track fires. The first provided the most years of data. The others ran for fewer years, detected more fires, and allowed us to test whether data source affected our results.

We tracked fire patterns with monthly composites of nighttime 1-km^2^ resolution hot pixels from the European Space Agency's Ionia World Fire Atlas (WFA) [42]. The WFA provides the longest running data set of global, active fire observations [43]. For 1996–2002, hot pixels are from the Along Track Scanning Radiometer (ATSR; ERS-2 satellite), and for 2003–2006, they are from the Advanced Along Track Scanning Radiometer (AATSR; Envisat satellite). The WFA sensors use 2 distinct temperature-threshold-based algorithms to detect hot pixels. We used hot pixels detected with the more sensitive Algorithm 2. It purports to detect a fire of 0.1 ha if it is hotter than 327°C. Because understory fires are rarely detected by satellites, and savanna and agricultural fires generally reach their hottest temperatures during the afternoon, we can safely assume that these nighttime detections represent hot deforestation fires. The overpass interval at the equator is 3 days and geo-location errors generally average 2–3 km [43].

Detecting fire from space remains challenging and each sensor has advantages and disadvantages. Detection algorithm, overpass time and frequency, spatial resolution, land cover, and type of fire all affect which fires are detected [20]. Stolle et al. [44] compared 8 different hot pixel data sets over the same area and time period. The datasets largely detected different fires and they were not complementary. Given these difficulties, we chose a long-running dataset, which provides one systematic look at patterns of deforestation fires over large spatial and temporal scales. To register the greatest number of fires possible, while avoiding commission errors, we used screened data from Mota et al. [43]. They removed errors caused by hot surfaces, gas flares, volcanoes, and sensor irregularities from Algorithm 2 of the WFA data. Omission errors are still a cause for concern. The satellite passes at night, so short-duration afternoon fires, such as burning pastures are not registered. The WFA data pick up the nighttime remains of hot deforestation fires, but miss fires burning beneath a forest canopy. Even if the sensor registered all fires, the overpass interval of 3 days ensures that many are missed. Because we use yearly composites, seasonal variation in cloud cover (which may also prevent fire detection) is not a major concern. The resulting data provide a systematic sample, albeit an underestimate, of Amazon fires.

To confirm the general pattern of our results, we also analyzed 3-year data sets released by the Large-Scale Biosphere-Atmosphere Experiment (LBA) in Amazônia [45]. The data are 2001–2003 hot pixels from 2 sensors: the Advanced Very High Resolution Radiometer (AVHRR) on NOAA-12 [46] and the Moderate Resolution Imaging Spectroradiometer (MODIS) on the Terra satellite. Like the WFA, these data have a resolution of 1 km^2^, but these satellites have daytime and more frequent overpass times. They detect more fires than the WFA. In addition to analyzing the full ten years of WFA data, we also separated 2001–2003 WFA data (denoted ATSR hereafter, although 2003 is from the AATSR sensor) and directly compared those years with the AVHRR and MODIS data.

Because of rapid change in land cover over the large spatial and temporal scale of our study, we did not include detailed land cover data. Instead, we assigned designations of forest or savanna vegetation derived from ecoregions [47]. Our forest designation included humid tropical forest, flooded forest (varzea and igapó), seasonal dry forest, and white-sand areas (campinas and campinaranas). We excluded the cerrado of the southwestern Amazon, lavrado of Roraima, savannas of Pará and Amapá, and a small area of Pantanal in Mato Grosso. Reserves with portions of these ecoregions within their borders were clipped to exclude them.

Social and economic drivers of fire and deforestation, as well as environmental variables, vary across the Amazon [26], [33]. State lines broadly reflect these differences. We used states and a geographic feature (the Xingu river) to divide the Legal Amazon into 2 regions, high-human-impact and low-human-impact (hereafter referred to as high-impact –HI– and low-impact –LI–, respectively), which we analyzed separately. The forests of Acre, Amazonas, Roraima, and Amapá are among the least disturbed in Brazil, with approximately 12%, 2%, 5%, and 2% deforestation, respectively, as of 2006. We designated these states as low-impact areas. Rondônia, Mato Grosso, Tocantins, and Maranhão had approximately 38%, 38%, 74% and 45% deforestation, respectively, in 2006. We designated these states as high-impact areas (area deforested and remaining forest in 2006 from http://www.dpi.inpe.br/prodesdigital/prodesmunicipal.php, accessed January 31, 2008). Pará had approximately 19% deforestation in 2006, but the majority of that deforestation occurred east of the Xingu River. Therefore, areas in Pará east of the Xingu River we classified as high-impact and the areas north and west of the Xingu River we considered low-impact.

We grouped reserves into fully protected parks (e.g., biological or ecological reserves, state and national parks), limited-use areas (e.g., national forests, extractive reserves, sustainable development reserves, state forests, and state environmental protection areas), and indigenous lands, on the basis of activities that they allow.

World Wide Fund for Nature-Brazil compiled the shape files of reserves. The original sources were FUNAI (Fundação Nacional do Índio; Indigenous reserves), IBAMA (Instituto Brasileiro do Meio Ambiente e dos Recursos Naturais Renováveis; federally protected areas), and the state secretaries of the environment (state protected areas). We excluded marine and mangrove reserves. To avoid co-registration errors, we excluded reserves of <100 km^2^ unless they were adjacent to another reserve of the same type. To avoid double-counting areas that had two different designations, we excluded limited-use areas and protected parks that overlapped by more than half of their area with indigenous lands. Altogether, we included the forest ecoregion portions of 53 parks, 109 limited-use reserves, and 238 indigenous reserves, totaling 180,125 km^2^, 409,984 km^2^, and 936,819 km^2^, respectively. The combined area was 1,526,928 km^2^ or approximately 37% of the forest ecoregion area of the Brazilian Amazon. We used road data and the Legal Amazon boundary from IBAMA (http://siscom.ibama.gov.br/shapes/; modified February 6, 2007, accessed April 30, 2007). Road data include state and federal roads, and some private roads, but omit many unofficial roads that are visible in Landsat Images. As the vast majority of hot pixels detected (∼90%) were ≤10 km of roads in our dataset, this omission should not significantly affect our results.

### Data analyses

For each 1-km^2^ pixel in forest ecoregions, we recorded the distance to the nearest road and whether the pixel had burned in a given year. We used analysis of covariance to assess patterns of hot pixel frequency in different reserve types, land designations (inside and outside reserves, high- and low-impact areas) and distance to roads (binned to 10 km wide classes). We measured ENSO severity with the Multivariate ENSO Index (MEI) [48] and compared numbers of hot pixels in a given year with that year's average MEI value.

The statistical analyses raise the issue of the independence of individual fires. Treating fires as independent observations would result in huge sample sizes. The sensors detect distinct fires — or clusters of fires — to the resolution of a 1-km^2^ pixel. However, hot pixels are typically clustered at a scale of a few square kilometers (possibly the scale at which individual ranches set fires). In any case, individual hot pixels were not independent observations. Consequently, we used regression analysis on the average number of hot pixels/100 km^2^, in each road distance class, and for each category (e.g., high-impact or low-impact, inside or outside of reserves). Here, the sample sizes were far smaller, but only the residuals about the model needed to be independent. At this scale, there is no reason to think that the residuals would be correlated. We restricted analyses to distance classes for which the total combined number of hot pixels in the 2 classes being compared (e.g., inside and outside reserves) was >50. Classes with <50 hot pixels were generally either very far from roads (very few hot pixels in huge remote areas) or had very little land area (high impact classes that covered almost no area and thus registered few pixels).

## Results

**Figure 1:**
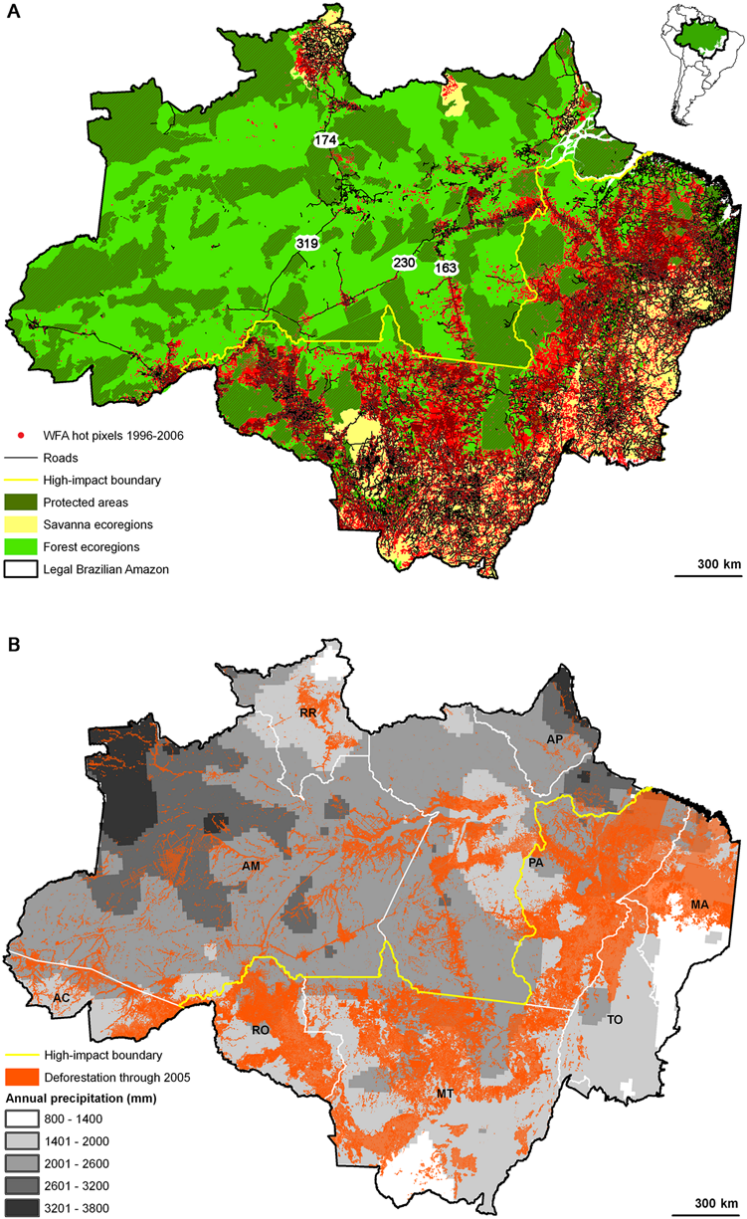
Fire and deforestation in the Brazilian Amazon. A) The Legal Brazilian Amazon showing reserves and World Fire Atlas hot pixels from 1996–2006. The high-impact forest is to the southeast and low-impact forest is to the northwest of the yellow boundary line. Roads mentioned in the text are labeled. B) PRODES deforestation polygons through 2005 against the background of annual rainfall from the WorldClim dataset. High-impact areas include the states of Rondônia [RO], Mato Grosso [MT], Tocantins [TO], Maranhão [MA] and the portion of Pará [PA] east of the Xingu River. Low-impact areas include the states of Acre [AC], Amazonas [AM], Roraima [RR], Amapá [AP] and the portion of Pará north and west of the Xingu river.

**Figure 2:**
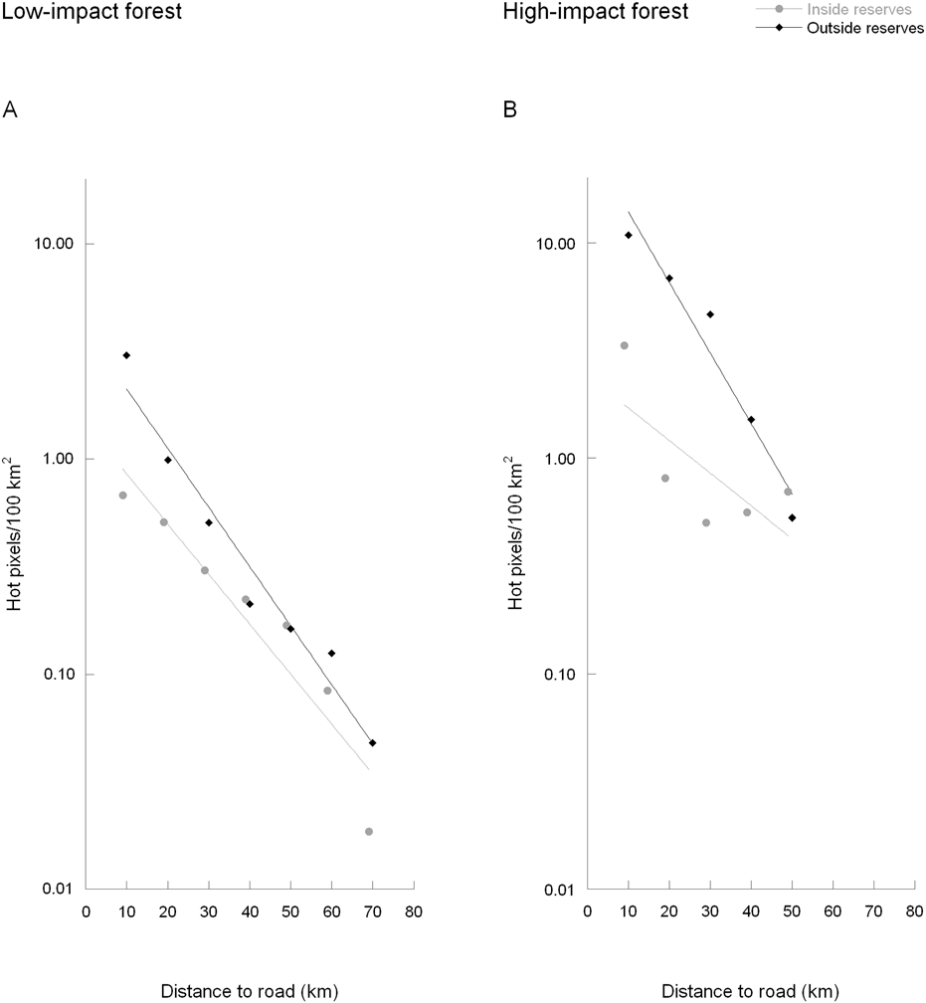
Relationship between 1996–2006 hot pixels/100 km^2^ and their distance to roads. A) low-impact and B) high-impact forests (low- and high- impact areas as shown in Fig. 1). Data are separated by whether fires are inside (grey) or outside (black) reserves. Fire rates were calculated on the basis of distance classes, but data points are offset from the class number for clarity (e.g., x values of 9 and 10 for class 10).

**Figure 3:**
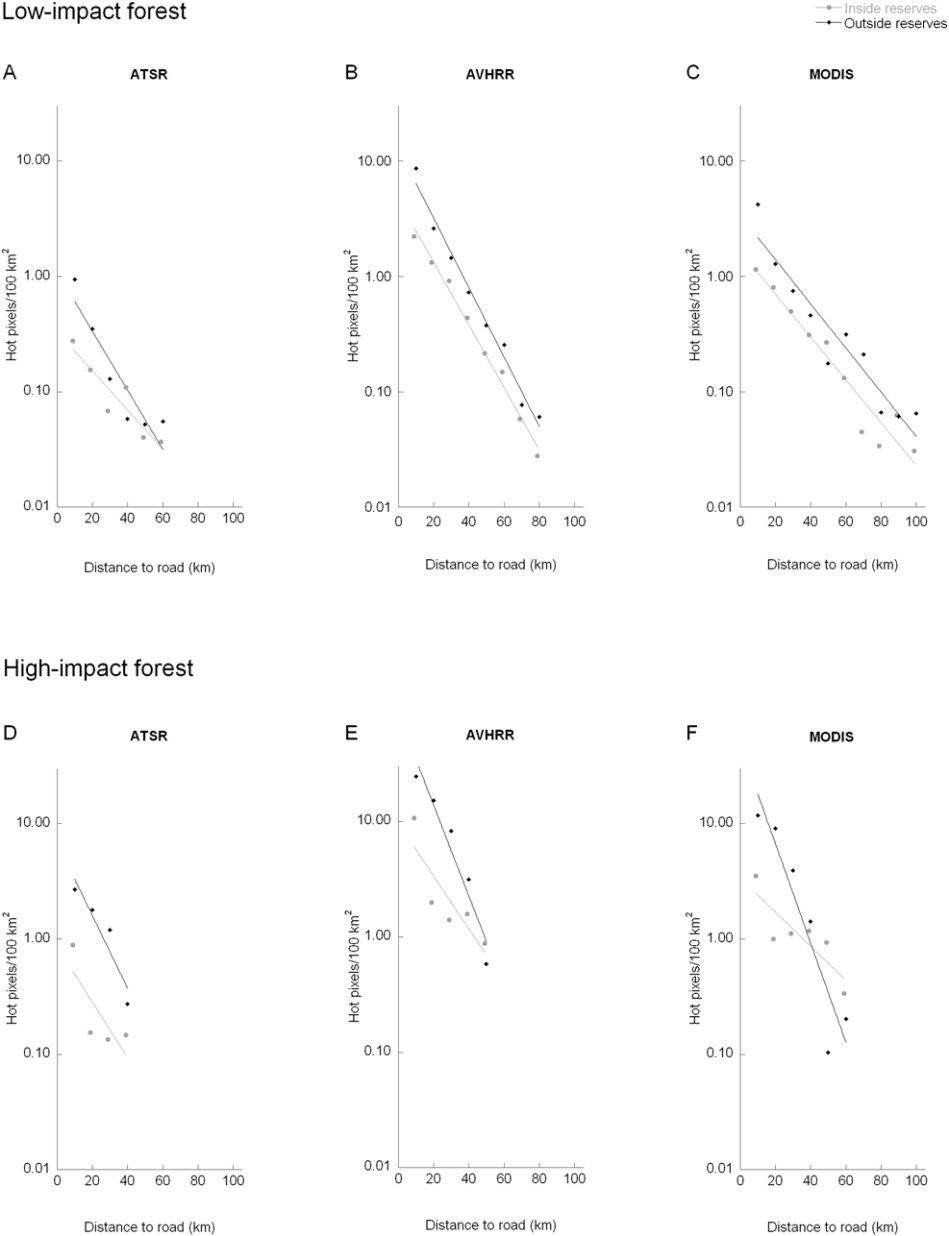
Relationship between 2001–2003 hot pixels/100 km^2^ and their distance to roads for 3 different sensors, ATSR/AATSR, AVHRR, and MODIS. A–C) low-impact and D–F) high-impact forests (low- and high- impact areas as shown in Fig. 1). Data are separated by whether fires are inside (grey) or outside (black) reserves. Fire rates were calculated on the basis of distance classes, but data points are offset from the class number for clarity (e.g., x values of 9 and 10 for class 10). All sensors detect at a 1-km^2^ resolution, but differ in detection algorithms and overpass times.

As expected based on state deforestation statistics, the majority (88%) of hot pixels detected in forest ecoregions with a decade of WFA data were in high-impact forest (Fig. 1) Only 12% of these deforestation fires were in low-impact forest. Almost 90% were ≤10 km from roads. (A few roads had no hot pixels, such as the unpaved and frequently impassable BR-319.) In both low- and high-impact forests (LI and HI), inside and outside reserves (Res and Out), there were compelling exponential declines in hot pixel frequency with increasing distance from roads (Fig. 2, 3). (That is, log fire incidences decreased linearly with distance and the linear relationships were good fits.) Although AVHRR and MODIS detect many more hot pixels than the WFA sensors, the exponential patterns of decline with distance from roads for AVHRR and MODIS were similar to the WFA data for the same years (Fig. 3; WFA 2001–2003 data denoted as ASTR). The relationships were significant at p<.05 for all sensors, inside and outside reserves, and in high- and low-impact areas (Tables 1, 2, row 1). Prior hypotheses expected declines, so the appropriate tests were one-tailed.

**Table 1:** Significance values for analysis of covariance tests of the patterns of decline in hot pixels with road distance inside and outside of reserves in Fig. 2 (WFA) and Fig. 3 (sensor comparison).

Data set^b^								
Test^a^	WFA LI	WFA HI	ATSR LI	ATSR HI	AVHRR LI	AVHRR HI	MODIS LI	MODIS HI
Distance	<0.0001*	<0.002*	<0.0001*	<0.02*	<0.0001*	<0.001*	<0.0001*	<0.0001*
Difference Res/Out	<0.01*	<0.004*	0.05*	<0.01*	0.0002*	<0.02*	<0.003*	0.17
Interaction Dist/Diff	0.17	<0.06	<0.1	0.33	<0.02*	<0.07	0.42	<0.01*

a Tests are: the decline of hot pixels with distance from roads (Distance), the difference between the numbers of hot pixels inside and outside reserves (Difference Res/Out) and the interaction between these 2 factors (Interaction Dist/Diff).

b Hot pixels are grouped into high-impact forest (HI) and low-impact forest (LI) and are from the following data sets: World Fire Atlas (ATSR/AATSR sensors) 1996–2006 (WFA), the World Fire Atlas (ATSR/AATSR sensors) for 2001–2003 (ATSR), and the Vegetation Fire Dynamics data set, including NOAA12 AVHRR 2001–2003 (AVHRR) and MODIS Terra 2001–2003 (MODIS). Values of one-tailed tests are marked with an asterisk (*) at a significance level of p<.05.

**Table 2:** Significance values for analysis of covariance tests of the differences between high-impact and low-impact forests (e.g., reserves in high-impact vs. reserves in low-impact) shown in Fig. 2 (WFA) and Fig. 3 (sensor comparison).

Data set^b^								
Tests^a^	WFA Res	WFA Out	ATSR Res	ATSR Out	AVHRR Res	AVHRR Out	MODIS Res	MODIS Out
Distance	0.0007*	<0.0001*	0.004*	0.0008*	<0.0001*	<0.0001*	<0.0001*	<0.0001*
Difference LI /HI	<0.003*	<0.0001*	0.11	<0.003*	0.0004*	0.0003*	0.0003*	0.44
Interaction Dist/Diff	0.17	0.15	0.26	0.30	0.22	0.04*	0.21	<0.002*

a Tests are: the decline of hot pixels with distance from roads (Distance), the difference between the numbers of hot pixels in low- and high-impact areas (Difference HI /LI) and the interaction between these two factors (Interaction Dist/Diff).

b Hot pixels are grouped into those inside (Res) and outside (Out) of reserves and are from the following data sets: World Fire Atlas (ATSR/AATSR sensors) 1996–2006 (WFA), the World Fire Atlas (ATSR/AATSR sensors) 2001–2003 (ATSR), and the Vegetation Fire Dynamics data set, including NOAA12 AVHRR 2001–2003 (AVHRR) and MODIS Terra 2001–2003 (MODIS). Values of one-tailed tests are marked with an asterisk (*) at a significance level of p<.05.

There were far fewer fires inside reserves than outside for both low- and high-impact forests (significant at p≤.05 for all sensors and areas except MODIS 2001–2003 high-impact; Table 1, row 2). Prior hypotheses also expected these differences, so the appropriate test was one-tailed.

The differences between reserves and outside reserves were generally greatest closest to roads. We tested this by examining whether a model with two regression slopes (inside versus outside) improved the statistical fit over a model with a common slope. We expected that at large distances from roads, it should matter less whether or not forest was inside a reserve; so again, the test was one-tailed. These results were mixed: two results were significant at p<.05, two more were close, but all differences were in the expected direction (Table 1; row 3).

Converging regression lines imply that there is some distance from roads beyond which there is no difference in fire frequencies between areas inside and outside of reserves. Treating each distance class as a separate variable in an ANOVA allowed us to ask at what distance from roads were fire frequencies statistically different inside versus outside reserves. For the eight sets of results in Table 1, those distances were 10 km (once), 20 km (5 times), and 30 km (twice). These are somewhat smaller distances than those where the regression lines intersect, but estimates of that intersection have very large confidence intervals.

In addition, there were more fires (inside and outside of reserves) in high-impact than in low-impact forests (Figs. 2, 3) and these differences were significant for all but 2 sensors and areas (Table 2, row 2). The differences between high- and low-impact areas with increasing road distance were significant in only 2 cases (Table 2, row 3). This may reflect small sample sizes, especially in high-impact forest, where there is little land >30 km from roads.

**Figure 4:**
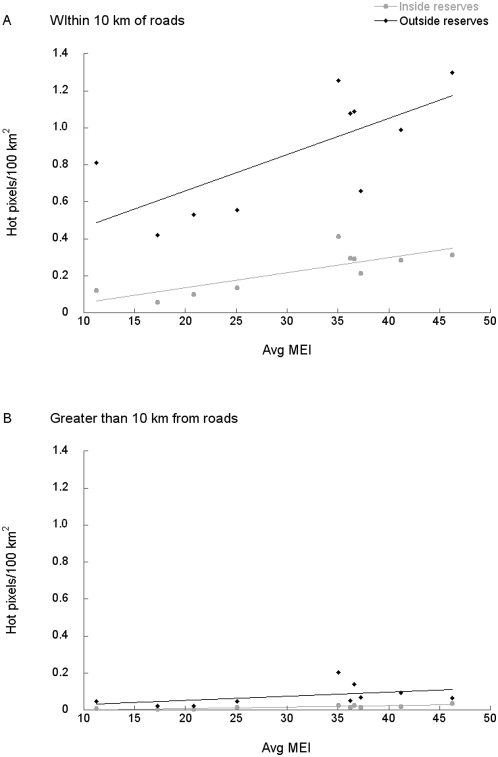
Relationship of the Multivariate ENSO Index (MEI) and the incidence of hot pixels/100 km^2^. Panels show (WFA, 1996–2005) hot pixels per year and the average yearly MEI A) within 10 km of roads (close) and B) more than 10 km from roads (far). Data are separated by whether fires are inside (grey) or outside (black) reserves. Significance values for analysis of covariance tests are as follows: inside reserves, close to roads: p<0.004; inside reserves, far from roads: p<0.004; outside reserves, close to roads: p<0.02; outside reserves, far from roads: p = 0.17.

There were generally more hot pixels in years with high ENSO indices than in years with lower ones. This was true both close (<10 km) and far (>10 km) from roads and inside and outside reserves (inside and close: p<0.004, inside and far: p<0.004, outside and close: p<0.02, only outside and far is not significant: p = 0.17; Fig. 4). As expected, there were more hot pixels near roads than far from them and more outside reserves than inside. These data were for both low- and high-impact forests analyzed together. There were too few data in each group to present low-impact forests separately. There was a numerically small, but statistically significant, increase in hot pixels inside reserves at >10 km from roads in high ENSO index years. This suggests that drought-stress may increase the likelihood of (probably already disturbed) forests being ignited, even far from roads. Close to roads and outside reserves, hot pixels increased dramatically with the drier conditions of a high ENSO index.

**Figure 5:**
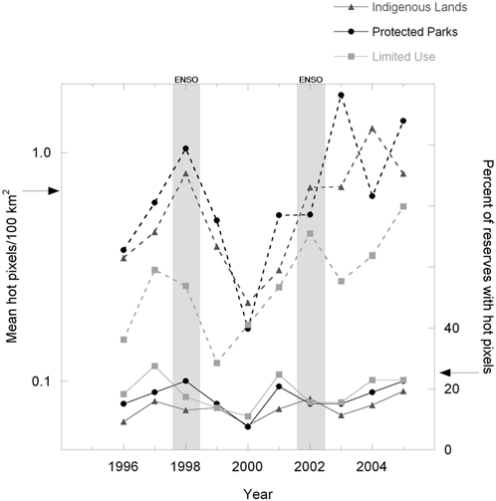
Differences in fire frequencies between fully protected parks, indigenous lands, and limited-use areas in the Brazilian Amazon. Solid lines (right axis) show the percentage of each reserve type (each year) with at least 1 hot pixel. Dashed lines (left axis) show the average number of hot pixels/100 km^2^ in those reserves that do have at least 1 hot pixel. Grey stripes indicate ENSO years.

**Figure 6:**
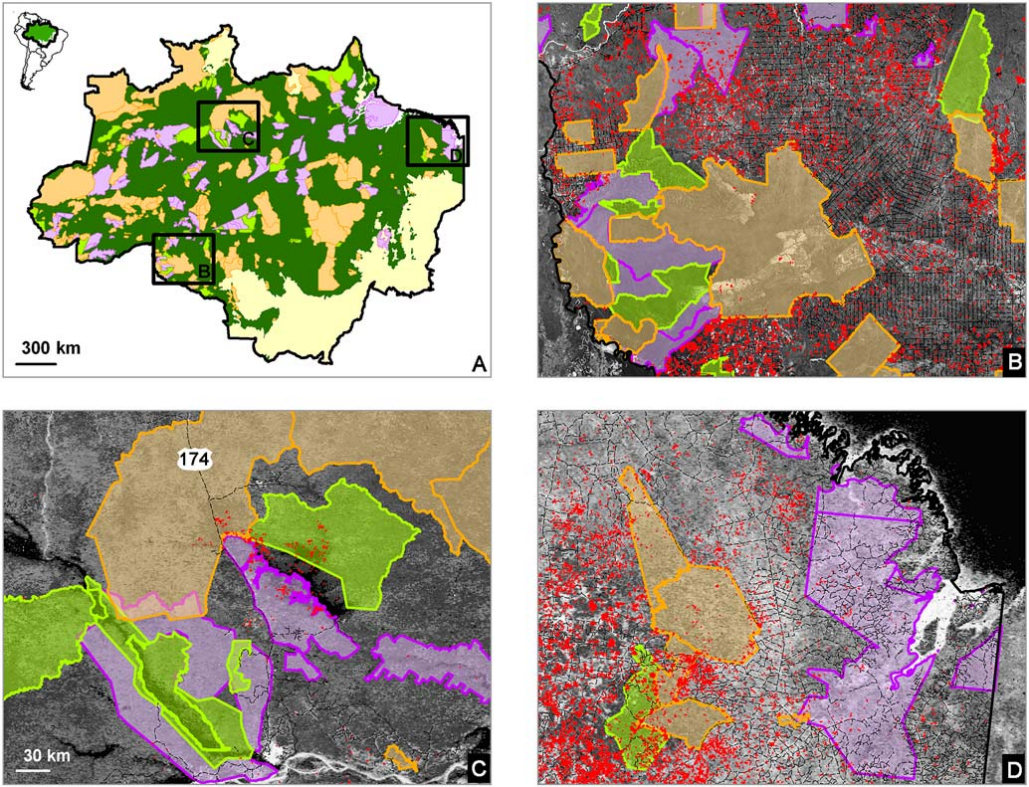
Regional differences in reserve protection against fire. A) Spatial distribution of reserves in the Brazilian Amazon. Close ups of areas in black squares where all reserve types are in close proximity, left to right: B) Rondônia, C) the BR-174 north of Manaus, and D) eastern Amazon (Maranhão and Pará). The WFA hot pixels for 1996–2006 are shown as red dots. Line colors denote reserve types: orange, indigenous lands; purple, limited use areas; green, fully protected parks. The background images are Landsat MrSID images (https://zulu.ssc.nasa.gov/mrsid/), and MODIS Blue Marble images, from the year 2000.

Reserve type had no significant effect. Most reserves (70–90%) had no hot pixels in any given year. In reserves with hot pixels, the average number/100 km^2^ generally varied together in all three types, with more hot pixels in ENSO years (Fig. 5). A slightly larger fraction of limited-use areas had fires (Fig. 5, bottom data series). Paradoxically, for reserves that did have fires, limited-use areas had slightly fewer fires/100 km^2^ (Fig. 5, top data series). There was a slight increasing trend in average hot pixels/100 km^2^ in all reserves over the ten-year period. The extent to which reserves of different types prevented fire depended largely on regional factors (Fig. 6) as we will discuss later.

## Discussion

### Reserves prevent fires, but it depends on where they are

Our first question was whether reserves actually protect Amazonian forests from deforestation fires. Our analysis clearly shows that they do. There are caveats, however, that relate to the second question of whether that protection is *de facto* or *de jure*.

Reserves had many fewer fires than areas outside, but protection differed between high- and low-impact areas. Overall, there are roughly 3 times more deforestation fires in high- than in low-impact areas. These regional differences have been obvious since at least the early-1970s [49], [50]. Inside reserves, fires were 4 times more frequent in high- than in low-impact areas. In addition to regional factors mentioned earlier (e.g., dry season length, agricultural practices, forest fragmentation, human density), this was likely due to the amount of reserve area close to roads. In low-impact forest, only about 5% of reserve area was ≤10 km from roads, compared with 20% of the area outside of reserves. In high-impact forests, 30% of reserve area was ≤10 km from roads, compared with 85% of the area outside reserves.

These differences illustrate the differences in pressure on reserves in high-impact areas. Even correcting for greater area outside reserves, there were always consistently more hot pixels close to roads outside reserves than inside, in both low- and high-impact areas. This difference diminished with increasing road distance. Because hot pixels are a proxy for deforestation, fewer fires close to roads inside reserves may relate to a lack of available infrastructure or to protected status that discourages land uses conducive to deforestation and fire along roads. This suggests that reserves provide the greatest protection from fires where the likelihood of burning would otherwise be greatest, that is, close to roads. On the other hand, the difference between fire occurrence in high- and low-impact reserves also indicates that reserves may not always provide sufficient protection when the pressure on them becomes very great. In addition, reserves that do not suffer deforestation fires may be subject to less detectable disturbance such as illegal logging or understory fire [51]. For example, some reserves in Acre that are known to have up to 6% of their area deforested [52] did not appear to have fires based on our data.

### Reserve type appears not to matter

There is no simple answer to our third question of whether some types of reserves are universally more effective. At the scale of the Brazilian Amazon, reserve type did not significantly affect fire frequency for a given distance to roads and region.

Statistical issues made it difficult to deny any effect of reserve type, however. First, only ∼20% of reserves had any hot pixels in most years (Fig. 5). In high fire years, this rose to 30%, still a small sample, once other variables were considered. The overall incidence of deforestation fires per area did not differ consistently among reserve types in different years. Second, local factors and the geographical arrangement of reserves made comparison of reserve types difficult. For example, most limited-use reserves are in remote regions with few fires (Fig. 6a). This explains why the average fire per area in limited-uses reserves appears low in Fig. 5. In high-impact areas, limited-use reserves tend to be small, with many fires.

### Regional factors are important

To illustrate regional differences, we examined 3 places (Rondônia, along the BR-174 highway in Amazonas, and an area in the eastern Amazon – Maranhão and Pará) where all the factors discussed were roughly equal, but where all 3 kinds of reserves were adjacent to each other (Fig. 6). This would seem to offer the best chance of detecting effects of reserve type.

In Rondônia, a massively deforested area, the contrast between fires inside and outside reserves was striking (Fig. 6b). In the centrally located indigenous reserve, the few hot pixels occurred in naturally dry ecosystems (nonforest, visible as light spots on the image). Protected parks here also suffered few fires, but this was likely because indigenous reserves surrounded them. In the north, there were many fires within a limited-use area. This suggests that limited-use reserves are less effective than those “policed” by indigenous peoples. This finding was confirmed by Ribeiro et al. [53] who found that deforestation in indigenous lands in Rondônia remained close to zero between 1997 and 2004, but raised concern about state sustainable use areas subject to high deforestation rates. Of the ten most deforested reserves (>20% deforested), seven are no longer considered as protected areas by the state governments (and thus are not included in our dataset). According to Ribeiro et al., there has been no specific law, changing the status of these areas, illustrating the vulnerability of state protected areas to the vagrancies of local governments. Using similar methods as Nepstad [21], we also analyzed hot pixel rates in 10-km wide inner and outer buffers at reserve borders in Rondônia. All reserve types protected against fires at their borders. Fire incidence outside reserves was 4–9 hot pixels/100 km^2^. Inside it was generally <2 hot pixels/km^2^.

Along the BR-174 in Amazonas, large areas (likely trees killed by the flooding of Balbina reservoir) burned in 1997 (Fig. 6c). These fires affected all adjacent reserves. One, the Waimiri-Atroari Indigenous Land, was mostly fire free, except for this spillover. Increased deforestation since repaving of the BR-174 highway in 1997 has not affected fire frequencies along this stretch because the inhabitants have strict rules about outsider use of the road.

In the eastern Amazon, in an area with many fires, no reserve has successfully kept fires at bay (Fig. 6d). For example, the Gurupi Biological reserve, a protected park, has not stopped logging, agriculture, and accompanying fires from spilling over from surrounding areas [54]. Adjacent indigenous lands and limited-use areas also burn frequently.

These examples illustrate the importance of local factors to the success of any reserve in protecting forest [21], [55]. As Nepstad et al. [21] also noted, the lack of obvious differences among reserve types is important, and demonstrates the usefulness of any reserve as protection against fire and deforestation. Lack of law enforcement and land thievery of “empty” government lands in the Amazon is a huge challenge [12], [26]. A reserve provides one important protection – especially if there is local enforcement, such as indigenous peoples with legal tenure [29]. Smallholders may also benefit from the enforcement and protection provided by a reserve, as seen recently in Pará [55].

Our results imply that the prevalently-held view that uninhabited reserves are the best kind for conservation may not be so clear cut, especially in the context of rapid infrastructure development and deforestation in the Brazilian Amazon. The fact that there is not a significant difference in deforestation fires in inhabited versus uninhabited reserves provides an immediate policy implication. Indigenous lands contain 5 times the land area of fully protected parks and form the majority of protected land in highly contested areas [30]. Some limited-use areas, such as Acre's extractive reserves, are managed to preserve forest cover and provide local jobs [56]. Both these types of reserves have, in many cases, been designated because of fierce grassroots pressure from local people, a process still underway in some areas. In the state of Roraima, the still fiercely contested indigenous reserve Raposa Serra do Sol is a current example of indigenous inhabitants advocating reserve creation to safeguard their land and resources from powerful economic interests, with benefits for biodiversity conservation [57]. Inhabited reserves thus might provide effective and (in some cases) politically feasible alternatives to more destructive land uses along new and existing roads, especially in contested areas.

### Conclusions: Roads, Fire, and Policy

Debate about the Amazon's future has rightly focused on roads as one of the most important drivers of deforestation [12], [25]. Roads provide access and raise land values [24], but specific economic and political circumstances are also tightly coupled with deforestation [40]. In the last decade, rising global demand for pasture-fed beef and soy and changes in the value of the Brazilian Real have, respectively, raised and lowered deforestation rates in the Amazon [13] and have also been correlated with fire [11].

Although previous work has found correlations between ENSO and understory fires [18], an important result of our work is the strong correlation between ENSO and deforestation fires at these spatial and temporal scales. Deforestation fires, such as those we are detecting, are all human-ignited. The implication is that either people are burning more in dry ENSO years, or that fires are more likely to escape in these years (or a combination of these factors). Reports of landowners sustaining large losses from escaped fires during periods of drought [58], suggests that people might not knowingly choose to burn during severely dry years. Indeed, the work of Moran et al. [59] suggests that many landowners in the Amazon have very little access to reliable weather information, and rely mainly on memory and experience to determine whether conditions are safe for burning. If this is the case, improved access to information, fire safety training for rural land owners, and strictly enforced burn-bans during dry periods might make a significant difference in the number of deforestation fires occurring [59]. Many Brazilian institutions, both governmental and nongovernmental, have taken steps in this direction [60]. Efforts to monitor and disseminate information about drought and fire conditions in Acre in 2005 [61] provide an example. Predictable inter-annual and geographic variation in climate clearly influences fire occurrence and provides a basis for year-to-year fire protection planning in different locations.

Most deforestation and fires have occurred in drier parts of the Amazon, but these processes already accompany roads built into more humid forests (notably BR-163). Even along roads within their borders, and even during ENSO-related drought, reserves of all types reduced fires that closely accompany roads throughout the Amazon. New and existing reserves should thus be an integral part of the planning process to mitigate the environmental impacts of roads [11], [12]. Plans to build or pave roads should also consider novel reserve forms, such as the “road park” (estrada parque) used in the Pantanal [62]. When reserves are designed in conjunction with local people and their needs, they may provide both environmental and resource protection, while lending the political force necessary to back reserves when powerful interests target them for exploitation.

## Supporting Information

Abstract S1 Abstract in Portuguese
(0.03 MB DOC)

Abstract S2 Abstract in Spanish
(0.03 MB DOC)
