# Supplementary material for: Reserves Protect against Deforestation Fires in the Amazon
Source: PLoS One. 2009 Apr 8;4(4):e5014. doi: 10.1371/journal.pone.0005014 (PMC2660414; doi:10.1371/journal.pone.0005014)
Supplement: Abstract S1 — Abstract in Portuguese (0.03 MB DOC) [file pone.0005014.s001.doc]

**Resumo**

**Contexto**

As áreas protegidas são a maneira principal de conservar as florestas e a biodiversidade, mas a questão de se as áreas protegidas funcionam é ainda debatida. Na Amazônia, as queimadas estão intimamente ligadas ao desmatamento e assim podem servir como uma indicação da eficácia de reservas na proteção da floresta. Nesse estudo, examinamos se as reservas na Amazônia brasileira fornecem uma proteção eficaz contra o desmatamento e conseqüentemente contra as queimadas; se essa proteção é por causa de sua localização ou seu estatuto jurídico; e se alguns tipos de reservas são mais eficazes do que outros.

**Metodologia/Resultados Principais**

Outros estudos já mostraram que a maioria das queimadas na Amazônia ocorrem próximo a estradas e que ocorrem mais freqüentemente em anos de El Niño. Determinamos estas relações para as reservas e as áreas não-protegidas por meio de uma regressão de focos de calor detectados por satélite contra a distância das estradas, através de toda a Amazônia brasileira e por uma década com 2 secas relacionadas ao El Niño. As queimadas de desmatamento, quando medidas por focos de calor, declinaram exponencialmente com a distância das estradas em todas as áreas. Menos queimadas de desmatamento ocorreram dentro das áreas protegidas do que fora delas e a diferença entre áreas protegidas e áreas não-protegidas foi maior próximo a estradas. Portanto, as reservas foram especialmente eficazes em impedir queimadas onde há mais probabilidade de incêndios; mas não forneceram proteção absoluta. Mesmo dentro das reservas, a qualquer distância das estradas, houve mais queimadas de desmatamento nas regiões com impacto humano elevado do que naquelas com baixo impacto humano. O efeito do El Niño em queimadas de desmatamento foi maior fora de reservas e próximo a estradas. As reservas índigenas, as reservas de uso-sustentável, e as reservas de proteção integral, todas tiveram menos queimadas do que áreas não-protegidas, e não pareceram diferir em sua eficácia.

**Conclusões/Relevância**

Controlando o tempo, fatores regionais, e o clima, nossos resultados mostram que as reservas são uma ferramenta eficaz para limitar queimadas destrutivas na Amazônia.
