# Supplementary material for: Reserves Protect against Deforestation Fires in the Amazon
Source: PLoS One. 2009 Apr 8;4(4):e5014. doi: 10.1371/journal.pone.0005014 (PMC2660414; doi:10.1371/journal.pone.0005014)
Supplement: Abstract S2 — Abstract in Spanish (0.03 MB DOC) [file pone.0005014.s002.doc]

**Resumen**

**Contexto**

Las áreas protegidas son la manera principal de conservar los bosques y la biodiversidad, pero el debate sobre si dichas áreas funcionan o no continua vigente. En la Amazonía, los incendios están estrechamente ligados a la deforestación y por lo tanto pueden servir como un indicativo de la eficacia de las reservas en la protección de los bosques. En este estudio, evaluamos si las reservas en la Amazonía Brasileña proporcionan una protección eficaz contra la deforestación y por lo tanto, contra los incendios, si esa protección es por causa de su localización o su estatus jurídico, y si algunos tipos de reservas son mas eficaces que otros.

**Métodos/ Hallazgos principales**

Otros estudios ya mostraron que la mayoría de los incendios en la Amazonía ocurren cerca de las carreteras y que ocurren más frecuentemente en años de El Niño. Determinamos estas relaciones, para las reservas y las áreas no-protegidas, por medio de una regresión de focos de calor detectados por satélite contra la distancia de las carreteras, a lo largo de toda la Amazonía brasileña, por una década con 2 sequías relacionadas a El Niño. Los incendios de deforestación, medidos por focos de calor, declinaron exponencialmente con la distancia a las carreteras en todas las áreas. Menos incendios de deforestación ocurrieron dentro de las áreas protegidas que afuera de ellas, y la diferencia entre áreas protegidas y áreas no-protegidas fue mayor cerca de las carreteras. Así, las reservas fueron especialmente eficaces en impedir incendios en lugares que sabemos que tienen más probabilidad de quemas; pero no proporcionaron la protección absoluta. Inclusive dentro de las reservas, a cualquier distancia de las carreteras, se presentaron más incendios de deforestación en las regiones con impacto humano elevado que en regiones con bajo impacto humano. El efecto de El Niño en incendios de deforestación fue mayor fuera de las reservas y cerca de las carreteras. Las reservas indígenas, las reservas de uso-sostenible, y las reservas de protección integral, todas tuvieron menos incendios que áreas no-protegidas, y no parecieron diferir en su eficacia.

**Conclusiones/Significancia**

Tomando en cuenta el tiempo, factores regionales, y el clima, nuestros resultados muestran que las reservas son una herramienta eficaz para limitar los incendios destructivos en la Amazonía.
